# Bimetallic Cu/Fe MOF-Based Nanosheet Film via Binder-Free Drop-Casting Route: A Highly Efficient Urea-Electrolysis Catalyst

**DOI:** 10.3390/nano12111916

**Published:** 2022-06-03

**Authors:** Supriya A. Patil, Nabeen K. Shrestha, Akbar I. Inamdar, Chinna Bathula, Jongwan Jung, Sajjad Hussain, Ghazanfar Nazir, Mosab Kaseem, Hyunsik Im, Hyungsang Kim

**Affiliations:** 1 Department of Nanotechnology and Advanced Materials Engineering, Sejong University, Seoul 05006, Korea; supriyaapatil11@gmail.com (S.A.P.); jwjung@sejong.ac.kr (J.J.); shussainawan@gmail.com (S.H.); gnazir@sejong.ac.kr (G.N.); mosabkaseem@sejong.ac.kr (M.K.); 2 Division of Physics and Semiconductor Science, Dongguk University, Seoul 04620, Korea; akbarphysics2002@gmail.com (A.I.I.); hyunsik7@dongguk.edu (H.I.); hskim@dongguk.edu (H.K.); 3 Division of Electronics and Electrical Engineering, Dongguk University, Seoul 04620, Korea; chinnuchem@gmail.com

**Keywords:** bimetallic, metal–organic framework, nanosheets, binder-free film, urea-electrolysis, ultra-high current

## Abstract

Developing efficient electrocatalysts for urea oxidation reaction (UOR) can be a promising alternative strategy to substitute the sluggish oxygen evolution reaction (OER), thereby producing hydrogen at a lower cell-voltage. Herein, we synthesized a binder-free thin film of ultrathin sheets of bimetallic Cu-Fe-based metal–organic frameworks (Cu/Fe-MOFs) on a nickel foam via a drop-casting route. In addition to the scalable route, the drop-casted film-electrode demonstrates the lower UOR potentials of 1.59, 1.58, 1.54, 1.51, 1.43 and 1.37 V vs. RHE to achieve the current densities of 2500, 2000, 1000, 500, 100 and 10 mA cm^−2^, respectively. These UOR potentials are relatively lower than that acquired by the pristine Fe-MOF-based film-electrode synthesized via a similar route. For example, at 1.59 V vs. RHE, the Cu/Fe-MOF electrode exhibits a remarkably ultra-high anodic current density of 2500 mA cm^−2^, while the pristine Fe-MOF electrode exhibits only 949.10 mA cm^−2^. It is worth noting that the Cu/Fe-MOF electrode at this potential exhibits an OER current density of only 725 mA cm^−2^, which is far inconsequential as compared to the UOR current densities, implying the profound impact of the bimetallic cores of the MOFs on catalyzing UOR. In addition, the Cu/Fe-MOF electrode also exhibits a long-term electrochemical robustness during UOR.

## 1. Introduction

Recently, hydrogen has been highly considered as a sustainable alternative to traditional fossil fuels owing to its high energy density of ~120 MJ kg^−1^, which is almost three times higher than that of the commonly used diesel or gasoline. In addition, the combustion of hydrogen releases only water as a by-product, and is, therefore, eco-friendly [1,2,3,4]. Among the several hydrogen production techniques, the electrochemical water-splitting process is the emission-free green route for generating hydrogen with high purity [5,6,7,8,9,10,11,12,13,14]. Unfortunately, the efficiency of hydrogen production is severely hindered due to the sluggish oxygen evolution reaction process (OER) at the anode [15,16,17,18]. This leads to a significant energy depletion and high cost for hydrogen production via the electrochemical water-splitting route. As a result, highly efficient electrocatalysts offering OER at lower overpotentials, and even alternative anodic processes that could substitute the sluggish OER and generate hydrogen efficiently at an overall lower cell voltage are highly important and urgent [19,20,21,22,23,24,25].

Recently, the urea oxidation reaction (UOR) has attracted great attention in urea-based energy conversion technologies as it allows for the simultaneous production of hydrogen and the treatment of urea-rich wastewater [23,26,27,28,29,30]. Theoretically, the UOR needs a significantly lower thermodynamic potential of 0.37 V while the OER requires a minimum of 1.23 V. Hence, the UOR offers advantageous energy savings and elevation of energy efficiency in hydrogen generation via electrochemical water-splitting [31,32,33]. Nevertheless, the UOR actually suffers from slow reaction kinetics due to the complex six-electron-transfer involved mechanism and the diverse adsorption/desorption of reaction intermediates [34,35]. The investigation of highly active, stable, and high-performance electrocatalysts for UOR is, therefore, required to overcome the slow reaction kinetics on UOR. On the other hand, despite the demonstration of good electrocatalytic activity for OER and UOR by the state-of-the-art RuO_2_ and IrO_2_ electrocatalysts, their commercial implementation is limited by the high cost and scarcity of these noble metal-based compounds [36]. Recently, various transition metal-based materials, such as sulfides [37,38,39,40], oxides [10,41,42], selenides [43,44,45] and metal–organic frameworks (MOFs) [11,46] have been investigated with noteworthy catalytic performance for UOR. However, they hardly fulfill the requirement for practical implementation due to their high overpotential and low current densities [23,26].

It should be noted that almost all high-performance UOR catalysts reported previously have hardly presented a UOR current density higher than 500 mA cm^−2^ (Table S1). This is because the UOR polarization curves at higher polarization potential often show deviation and ultimately switch the reaction toward OER due to mass transfer limitations. Although very few works have presented the mass transfer limiting UOR current in the polarization curves, no relevant discussion on this point can be found [47,48,49,50]. The highly porous catalyst materials with an abundant surface area such as MOFs not only enhance the electrocatalytic activity but also can facilitate the detachment of the gas bubbles evolved at the catalyst surface promoting the diffusion of urea molecules to the catalytic active sites. Interestingly, the large diversity of metal ions and organic ligands link together to form large varieties of MOFs featuring an adjustable coordination mode and crystalline structure at the molecular level. This, thus, generates a highly porous and crystalline compound sparking a lot of interest in electrocatalytic applications [11,13,23,51,52,53]. Moreover, thin two-dimensional (2D) MOF films hold their extensively exposed high percentages of coordinatively unsaturated metal sites with dangling bonds that are particularly favorable for electrocatalysis [54,55].

Hence, to overcome the issue of poor mass transport during UOR, we designed a binder-free MOF-ink-based 2D-thin film on porous nickel foam (NF) substrate as a highly efficient UOR catalyst via a drop-casting approach. Notably, the bimetallic Cu/Fe-MOF electrode exhibited a remarkably ultra-high anodic current density of 2500 mA cm^−2^ only at 1.59 V vs. RHE, while, in contrast, the pristine Fe-MOF electrode exhibited only 949.10 mA cm^−2^ at the same bias. To the best of our knowledge, this is the first report on such an ultra-high current density in 0.33 M urea added 1.0 M KOH aqueous electrolyte.

## 2. Materials and Methods

### 2.1. Reagents and Materials

Reagent grade 2-aminoterephthalic acid (99%), copper (II) nitrate trihydrate (Cu (NO_3_)_2_·3H_2_O, 99%), iron (III) chloride hexahydrate (FeCl_3_·6H_2_O, ≥99%), acetic acid (≥98%), hydrochloric acid (37%), N, N-dimethylformamide (DMF, 99.8%), methanol (≥99.9%), acetone (≥99.9%) and potassium hydroxide (KOH, ≥85%) were purchased from Sigma-Aldrich (St. Louis, MO, USA). As a current collector supporting electrode, 1.6 mm thick nickel foam (NF) substrates were purchased from Alantum Corporation (South Korea). Through ultrasonic agitation, 1 × 5 cm^2^ pieces of the NF-substrates were washed for 10 min in 2 M HCl, deionized water, ethanol, and acetone. The washed substrates were dried in air at room temperature for 24 h.

### 2.2. Bulk MOF Powder Synthesis

The bulk powder of Cu/Fe-MOF was synthesized solvothermally as follows: 1.5 millimole FeCl_3_·6H_2_O, 0.75 millimole Cu(NO_3_)_2_·3H_2_O and 2.25 millimole 2-aminoterephthalic acid were dissolved in 50 mL *N*,*N*-dimethylformamide (DMF). To this mixture, 0.75 mL acetic acid was added. The mixture was then transferred to a Teflon-lined stainless-steel autoclave and reacted at 150 °C for 5 h. A similar procedure was used to synthesize the Fe-MOF and Cu-MOF by reacting FeCl_3_·6H_2_O (2.25 mmol) or Cu(NO_3_)_2_·3H_2_O (2.25 mmol) reacting separately with 2-aminoterephthalic acid (2.25 mmol) and acetic acid (0.75 mL) in 50 mL DMF at 150 °C for 5 h.

### 2.3. Drop Casting of MOF Films

For drop-casting the MOF films, MOF-inks were first prepared by ultrasonication of the solvothermally synthesized 100 µg MOF powder in 1 mL of DMF solution for 2 h at a frequency of 38.1 kHz in an ultrasonic bath. The freshly prepared ink was dropped into a NF-substrate slowly until the entire surface of the substrate became wet with the ink. Finally, the drop-casted films were dried at 60 °C overnight, and as an active geometrical surface area, 1 cm^2^ of the film-coated substrate was exposed using Teflon tape followed by pressing of the taped masking area of the NF-substrate via a stainless-steel twister.

### 2.4. Electrochemical Measurements

All electrochemical measurements were conducted in a homemade three-electrode cell system with an NF-based electrode serving as the working electrode while a graphite rod and a saturated calomel electrode (SCE) were employed as the counter and reference electrodes, respectively. All measurements were recorded using a BioLogic Science Instruments electrochemical workstation. The potentials were converted to the reversible hydrogen electrode (RHE) scale according to relation given below.

*E*_RHE_ = *E*_SCE_ + *E*°_SCE_ + (0.059) pH (1)

where *E*°_SCE_ is taken as 0.241 V, and pH is the measured pH of the aqueous 1.0 M KOH and 0.33 M urea added 1.0 M KOH electrolyte solution. 

The NF-based electrodes were first rinsed with 1.0 M KOH or 0.33 M urea added 1.0 M KOH solution before subjecting into the measurement cell. Prior to measurement, the working electrodes were conditioned via cyclic voltammetry (CV) at a scan rate of 100 mV s^−1^ until stable voltammograms were obtained. Linear sweep voltammogram (LSV) curves and CV were recorded at a potential sweeping rate of 5 mVs^−1^. Electrochemical impedance spectroscopy (EIS) was conducted in the same electrochemical working station. All voltammograms were recorded with an iR drop compensation.

## 3. Results and Discussion

### 3.1. Film Morphology and Crystal Structure

**Figure 1:**
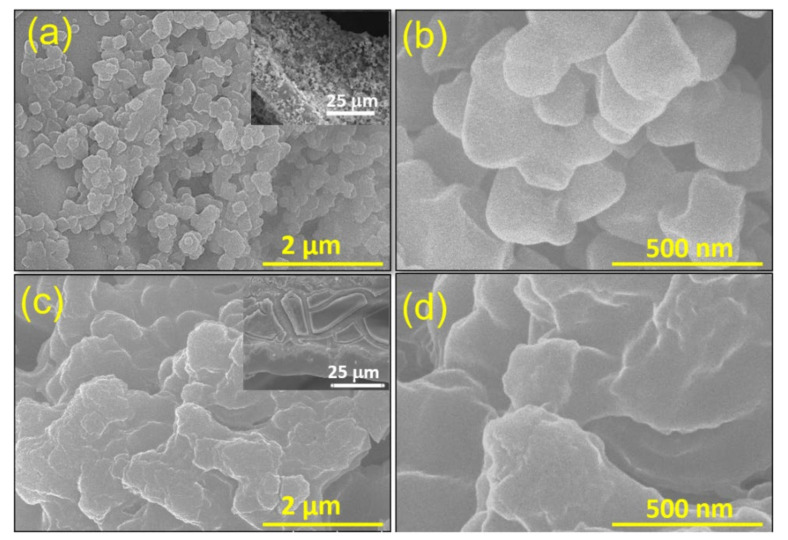
SEM images of the drop-casted (**a**,**b**) Fe-MOF and (**c**,**d**) Cu/Fe-MOF films on a nickel foam at various magnifications. Inset images are the lower magnified SEM views of the corresponding samples in microscale range.

The drop-casted MOF films on the NF-substrate appeared faint red. The surface morphology of the films was examined via a field-emission scanning electron microscope (FE-SEM). Figure 1a,b shows the SEM view of the Fe-MOF film on a NF-substrate. This film shows nano-particles (~300 nm) of MOF spreading uniformly on the entire surface of the substrate. In contrast, Figure 1c,d shows that the drop-casted Cu/Fe-MOF film is comprised of thin sheets. The large sheet-like structure deposited on a nickel backbone of the foam substrate is apparent in the inset image of Figure 1c and Figure S1 while the sheets are actually consisted of ultra-thin layered sheets, as shown in Figure 1d. Such thin film consisting of large nanosheets with lesser grain boundaries possesses a lower interfacial charge transfer resistance, thereby enhancing the output current.

**Figure 2:**
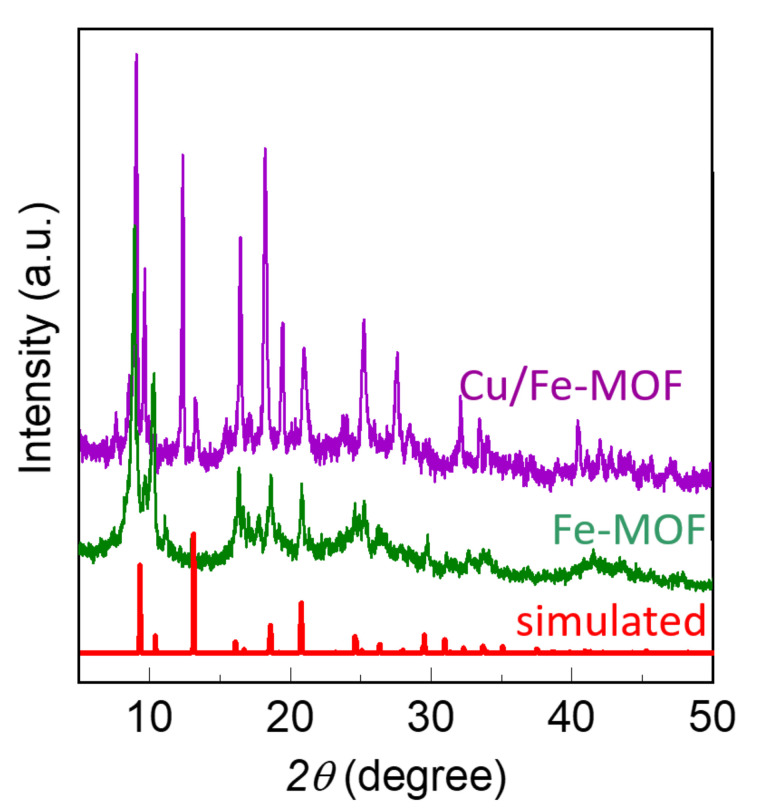
XRD patterns of the bulk powder MOFs and the corresponding simulated XRD patterns of the reference NH_2_-MIL-88 MOF.

The drop-casted film was extremely thin and was formed from the very dilute MOF-inks (100 µg/mL). As a result, the films did not exhibit X-ray diffraction (XRD) peaks. However, the MOF-powders from which the inks were prepared showed sharp crystalline XRD peaks, as displayed in Figure 2. Both the XRD patterns possess similar positions of their major peaks, suggesting that the Fe-MOFs and Cu/Fe-MOFs have similar crystal structures. Furthermore, the XRD patterns of these MOFs show resembling patterns to that of the NH_2_-MIL-88B family [56,57,58]. This finding suggests that both the MOFs have a crystal structure belonging to a hexagonal space group (P63/MMC) [59].

**Figure 3:**
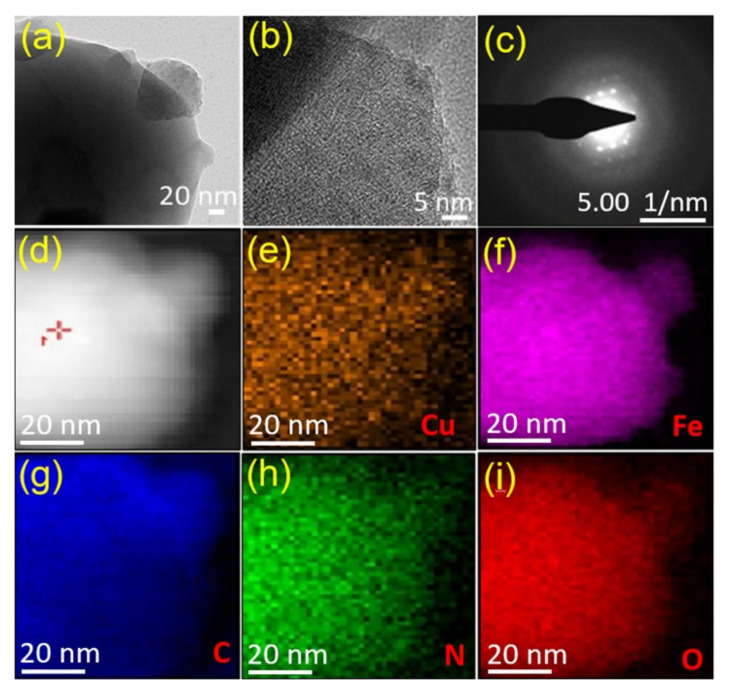
(**a**) TEM image, (**b**) HR-TEM image, (**c**) SAED pattern, (**d**) HAADF STEM image, and (**e**–**i**) element distribution mapping on a Cu/Fe-MOF nanosheet. Individual elements: Cu (orange), Fe (pink), C (blue), N (green), and O (red).

The surface morphology and crystal structure of the Cu/Fe-MOFs, which demonstrated an outstanding UOR performance compared to the Fe-MOFs, were further studied via transmission electron microscopy (TEM) and selected area electron diffraction (SAED) analysis. Figure 3a shows the TEM image of the Cu/Fe-MOFs, which, in line with the SEM finding, also reveals a thin-sheet morphology. In a high-resolution TEM mode, the organic ligand of the MOFs could hardly withstand the highly energetic focused electron beams. As a result, while focusing, the MOF crystals melted and the HR-TEM showed an amorphous structure (Figure 3b). However, with enormous effort, the SAED image exhibiting bright crystalline spots was obtained, as shown in Figure 3c. This finding indicates that the synthesized MOFs were actually crystalline, which is in good agreement with that of the XRD finding.

### 3.2. Chemical Composition and Binding State of the MOF-Film

**Figure 4:**
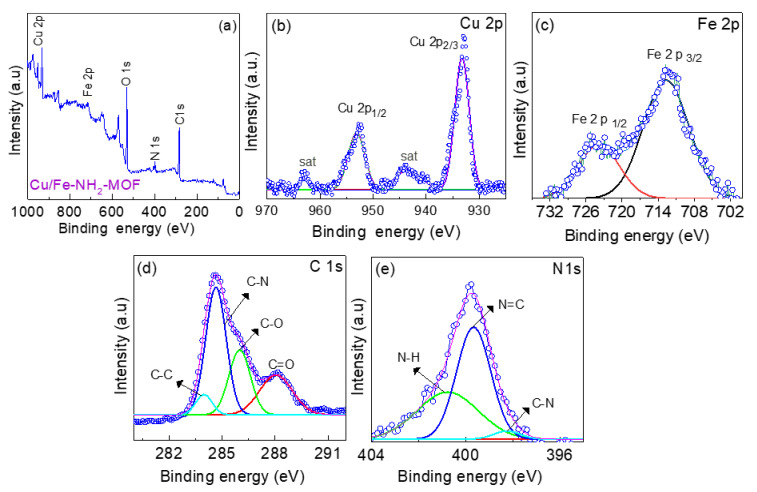
(**a**) XPS survey spectrum, and high-resolution XPS spectrum of (**b**) Cu 2p (**c**), Fe 2p (**d**) C 1s, and (**e**) N 1s of the drop-casted Cu/Fe-MOF film (Blue open circles represent experimental data and solid purple, red, green, blue and sky-blue lines represent corresponding fitting results).

To understand the chemical composition of the Cu/Fe-MOFs, X-ray photoelectron spectroscopy (XPS) was conducted. Figure 4a shows the elemental survey spectrum revealing the presence of Cu, Fe, O, N, and C as the main constituents of the MOFs. The high-angle annular dark-field (HAADF) imaging of the Cu/Fe-MOFs and the corresponding element mapping were also conducted via scanning transmission electron microscopy (STEM). The results are displayed in Figure 3d–i, which shows the uniform distribution of the above constitutional Cu, Fe, C, O, and N elements over the entire sheet of the MOFs. Furthermore, to gain insight into the chemical binding states, high-resolution XPS spectra of these constitutional elements were studied.

Figure 4b displays the Cu 2p XPS spectrum showing the two sharp doublets centering at ~953.08 eV and 933.22 eV. These peaks can be assigned to the Cu 2p_1/2_ and Cu 2p_3/2_ orbitals, respectively. In addition, a pair of associated satellite peaks centering at ~962.93 eV and 943.71 eV can also be observed. This finding indicates that the Cu ions that coordinated with the NH_2_-BDC ligand in the Cu/Fe-MOFs are in the 2+ oxidation state [60]. Likewise, the high-resolution Fe 2p XPS spectrum has a pair of peaks centering at ~713.59 eV and 725.02 eV, as shown in Figure 4c. These peaks can be assigned to the Fe 2p_3/2_ and Fe 2p_1/2_ orbitals, respectively. This finding indicates that the Fe ions in the Cu/Fe-MOFs are in the 3+ oxidation state [58]. Moreover, the C 1s spectrum (Figure 4d) shows the presence of C–N bond from the –NH_2_ moiety, and the C=O bond from the carboxyl moiety linked to the benzene ring of the organic ligand. Similarly, the N 1s spectrum also shows the presence of C-N bonding (Figure 4e) [61,62]. These findings imply that the Cu and Fe ions are coordinated with the amino-terephthalic acid ligand forming the bimetallic Cu/Fe-MOFs [13].

### 3.3. Electrocatalytic Activity toward UOR and HER

**Figure 5:**
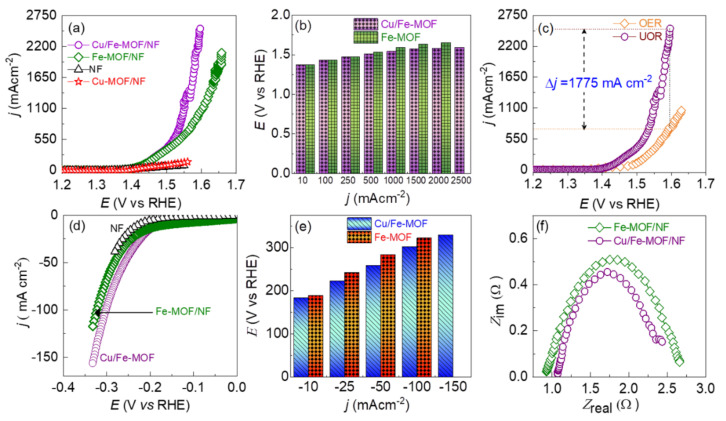
(**a**) Anodic polarization curves in 1.0 M KOH containing 0.33 M urea. (**b**) Corresponding comparison potential vs. UOR current density-based bar graph extracted from the anodic LSV. (**c**) Comparison of the OER and UOR polarization curves for the Cu/Fe-MOF. (**d**) Cathodic polarization curves. (**e**) Corresponding comparison potential vs. HER current density-based bar graph extracted from the cathodic LSV curves. (**f**) Nyquist plots from the EIS data.

To evaluate the electrocatalytic performance of the drop-casted MOF films on NF-substrate, the MOF-film-based electrodes were polarized in 0.33 M added 1.0 M KOH aqueous electrolyte against a SCE reference electrode and a graphite counter electrode. To assess the UOR performance, the electrodes were polarized anodically and the obtained LSV polarization curves are displayed in Figure 5a. The bare NF-substrate shows almost no catalysis on UOR. However, the drop-casted MOF films on the NF-substrate exhibited a distinctly high current revealing the noteworthy catalysis on UOR. From the difference in polarization curves of the bare NF-substrate and drop-casted Cu/Fe- and Fe-MOF films on the NF-substrate, it can be ratified that the UOR current density solely corresponds to the MOFs-films. However, it should be noted that the Cu-MOF film exhibited a poor catalytic activity similar to that of the bare NF. As a consequence, no further studies on the Cu-MOF film were conducted. Interestingly, the LSV polarization curves show that both the Cu/Fe-MOF/NF and Fe-MOF/NF electrodes have similar UOR activity up to 1.49 V vs. RHE where the UOR current density is ~350 mA cm^−2^. However, beyond this point, the Cu/Fe-MOF/NF electrode exhibited a higher catalytic activity on the UOR. Specifically, this electrode showed 2500, 2000, 1000, 500, 100 and 10 mA cm^−2^ at 1.59, 1.58, 1.54, 1.51, 1.43 and 1.37 V vs. RHE, whereas the Fe-MOF/NF electrode showed 2000, 1500, 1000, 500, 100 and 10 mA cm^−2^ at 1.65, 1.59, 1.53, 1.43, and 1.37 V vs. RHE, respectively. In addition, in order to minimize the influence of the high surface area (in a given geometrical surface area) of the porous NF substrate on the UOR performance, the ECSA (electrochemically active surface area) specific UOR activity was also determined by normalizing the current density by the ECSA. The result is displayed in Figure S2, which in line with the geometrical surface area-based LSV polarization curves (Figure 5a) reveals that the bimetallic Cu/Fe-MOF/NF electrode has superior UOR catalytic activity compared to the Fe-MOF/NF electrode. The observed catalytic activity of the Cu/Fe-MOF/NF electrode is attributed to synergistic interplay by the mutual coordination of Cu and Fe metal ions with the organic, providing a favorable environment for the UOR. The detailed UOR current density vs. electrode potential profile is shown in Figure 5b. As discussed earlier, regardless of the bias potential, the majority of the high-performance UOR catalysts have presented UOR current density up to about 500 mA cm^−2^ (Table S1). This could be the tricky way of data presentation hiding the mass transfer limited UOR current density [47,48,49,50]. It is worth noting that both the MOF-film-based electrodes demonstrated an ultra-high UOR current density beyond 2000 mA cm^−2^ without deviation in the LSV polarization curves caused by the mass transfer limitation. To the best of our knowledge, this is the first report of such a high UOR current density. The ultra-high UOR current density implies that the urea was decontaminated at a high rate through the anodic oxidation reaction on one hand, and a high rate of hydrogen production was achieved reciprocally on the other hand at the cathodic side.

In addition to the UOR, the OER performance of the Cu/Fe-MOF/NF was also tested briefly via LSV polarization in pure 1.0 M KOH aqueous electrolyte under the same conditions as applied for the UOR test. Figure 5c shows that the Cu/Fe-MOF/NF electrode has an onset potential of 1.37 V vs. RHE for the UOR while it is 1.41 V vs. RHE for the OER. In addition, the electrode achieved the OER current densities of 100, 500, and 1000 mA cm^−2^ at 1.49, 1.56, and 1.63 V vs. RHE, respectively, demonstrating that the UOR took place prior to the OER by 200 mV to attain the UOR current density of 100 mA cm^−2^. In addition, a large difference in catalytic activity between the UOR and OER can be observed particularly at high currents where the catalysis runs at high rates. For example, as can be seen in Figure 5c, the Cu/Fe-MOF/NF electrode at 1.59 V vs. RHE exhibited an ultra-high UOR current density of 2500 mA cm^−2^, while in contrast, the electrode exhibited an OER current density of only 725 mA cm^−2^, revealing a significantly large difference in current density of ~1775 mA cm^−2^. This finding, thus, implies that the sluggish OER can be replaced favorably by the UOR anodic reaction to generate hydrogen at a lower cell voltage. However, based on the closer onset potentials for both the UOR (1.37 V vs. RHE) and OER (1.41 V vs. RHE) processes, it should be noted that the Cu/Fe-MOF/NF anode was active for both the processes, which could possibly be a reason for the obtained ultra-high anodic current density at lower cell potentials. Nevertheless, a higher anodic current at a lower cell potential is always advantageous for the generation of hydrogen at a high rate at the cathode, which is the targeted goal in water-electrolysis. A slightly change in slope of the LSV curves of the Cu/Fe-MOF/NF electrodes can be observed in Figure 5a,c at about 1.55 V vs. RHE. This is due to the strong evolution of gas bubbles at the anode surface, which can be attributed to the simultaneous occurrence of the UOR and OER, decreasing the mass transport properties.

Furthermore, to access the catalytic activity on HER, the MOF-based electrodes were polarized cathodically in 0.33 M urea added 1.0 M KOH aqueous electrolyte, and the obtained cathodic LSV polarization curves are shown in Figure 5d. As in the case of UOR, the NF-substrate shows the poorest HER catalytic activity and the Cu/Fe-MOF/NF electrode has a superior HER catalytic activity than that of the Fe-MOF/NF electrode. The detailed HER overpotential (*η*) vs. current density profile is shown in Figure 5e. The reaction kinetics of the MOF-based electrodes toward UOR and HER were examined using Tafel slopes, as shown in Figure S3. A UOR Tafel slope of 27.5 mVdec^−1^ was determined for the Cu/Fe-MOF/NF electrode, which is much lower than that of the Fe-MOF/NF (31.9 mVdec^−1^), Fe-MOF/NF (34.3 mVdec^−1^) and bare NF-substrate (77.5 mVdec^−1^), highlighting that, in addition to the favorable nanosheet morphology having a lower number of grain boundaries, the coordination of Cu ions into Fe-MOFs also plays a key synergistic role in accelerating the UOR kinetics. The obtained low operation potential of the Cu/Fe-MOF/NF electrode is further compared with the recently reported UOR catalysts in Table S1 of the Supporting Information section. To explore the kinetics between the electrode and the electrolyte in greater depth, electrochemical impedance spectroscopy (EIS) was employed. Notably, the Cu/Fe-MOF/NF electrode exhibited a lower charge transfer resistance for UOR (~1.24 Ω) compared with the pristine Fe-MOF/NF electrode (~1.77 Ω), as shown in Figure 5f and Figure S4. In line with the UOR kinetics, the Cu/Fe-MOF/NF electrode also exhibited a relatively lower HER Tafel slope of 106.5 mVdec^−1^ than that of the pristine Fe-MOF/NF electrode (218.6 mVdec^−1^) (Figure S3b). To understand further the enhanced intrinsic UOR catalytic activity of the Cu/Fe-MOF/NF electrode, the electrochemically active surface area (ECSA) of the electrodes was evaluated. For this, the CV curves at various scanning rates ranging from 20 to 90 mV s^−1^ were recorded in 0.33 M added 1.0 M KOH electrolyte, and the double-layer capacitance (C_dl_) values were determined directly from the slope of the average current density (Δ*j*) vs. scan rate plots, as shown in Figure S5. The ECSA of the electrodes was estimated from the relation, C_dl_/Cs, where Cs is the specific capacitance of the electrode materials and is generally taken as 0.04 mFcm^−2^ for flat surface electrodes in 1.0 M KOH aqueous electrolyte [63]. Thus, the ECSA of the Fe-MOF/NF and Cu/Fe-MOF/NF electrodes was determined to be 77.75 and 89.75 cm^2^, respectively. The relatively larger ECSA suggests that compared to the pristine Fe-MOF/NF electrode, the Cu/Fe-MOF/NF electrode has higher number of catalytic active sites for the UOR.

**Figure 6:**
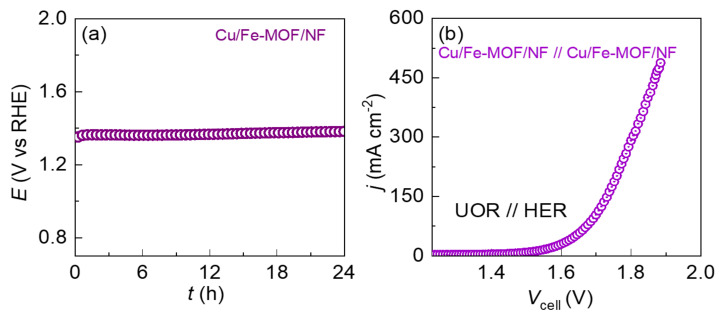
(**a**) Chronoamperometric stability test for the Cu/Fe-MOF/NF in the half-cell configuration system at a current density 10 mA cm^−2^ in 1.0 M KOH containing 0.33 M urea. (**b**) Polarization curves of overall urea oxidation electrolyzers with the electrode pairs: Cu/Fe-MOF/NF || Cu/Fe-MOF/NF in 1.0 M KOH containing 0.33 M urea.

Apart from the catalytic activity, the electrode should be electrochemically robust during a long-term UOR. Therefore, the electrochemical durability of the drop-casted MOF-films-based electrodes were evaluated chronopotentiometrically for 24 h against UOR at a standard bias of 10 mA cm^−2^. The chronopotentiometric stability test curves are shown in Figure 6a. It should be noted that despite the binder-free drop-casted films, both the MOF-film-based electrodes exhibited long-term robust stability toward UOR catalysis. It should be noted that the XRD of the drop-casted MOF-films on the NF before and after the UOR stability testing were identical showing only the XRD peaks from the NF-substrate. However, this could be due to the extremely thin MOF-film deposited from the very dilute MOF-ink (100 µg/mL). Hence, the possibility of the phase change of the film materials during the long-term UOR could not be ruled out.

The notable catalytic activity observed above for the UOR and HER encouraged us to explore the two symmetrical Cu/Fe-MOF/NF electrodes in a practical application, employing them as cathode and anode electrode materials of a urea electrolyzer at ambient temperature. Figure 6b displays an overall urea-splitting polarization curve of the electrolyzer. As can be observed from the polarization curves, the Cu/Fe-MOF/NF electrode-based electrolyzer exhibited a higher UOR current density at lower cell voltage (*V*_cell_), revealing an enhanced catalytic performance on overall urea-splitting. The details of the UOR current density vs. *V*_cell_ profile are shown in Figure S6.

## 4. Conclusions

A thin-film of MOF-nanosheets was deposited successfully on a nickel foam substrate via a drop-casting route using a very dilute (MOF leading: 100 mg mL^−1^) and binder-free MOF-inks at ambient temperature. Anodic and cathodic polarization of the MOF-film-based electrodes showed that the mutual coordination of the Cu and Fe ions with the organic linkers forming a bimetallic Cu/Fe-MOF exhibited a key synergistic interplay, providing a favorable morphological and electronical environment for the superior electrocatalytic performance on the UOR. The drop-casted Cu/Fe-MOF/NF electrode required only 1.59 V vs. RHE to yield a remarkably ultra-high anodic current density of 2500 mA cm^−2^ in 0.33 M urea added 1.0 M KOH aqueous electrolyte, while in contrast, the pristine Fe-MOF/NF electrode exhibited only a UOR current density of 725 mA cm^−2^ at the same bias. More impressively, the UOR catalytic activity remained stable for 24 h of continuous operational testing at the standard current density of 10 mA cm^−2^. This work paves the way for mass-scale production of noble-metal-free based ultra-thin films of MOF-nanosheets. These nanosheets represent promising robust and efficient electrocatalysts aimed at replacing OER with an alternative anodic UOR for the generation of hydrogen economically.

## Data Availability

The data presented in this study are available in this article and Supplementary Materials.

## Supplementary Materials

The following supporting information can be downloaded at: https://www.mdpi.com/article/10.3390/nano12111916/s1, Figure S1: SEM image of the drop-casted Cu/Fe-MOF film, Figure S2: ESCA specific UOR LSV polarization curves. Figure S3: Tafel slopes for UOR extracted from the corresponding anodic LSV polarization curves of “Figure 5a,b”. Figure S4: Electrochemical impedance spectroscopy. Figure S5: CV plots of the Fe-MOF/NF and Cu/Fe-MOF/NF electrodes and double-layer capacitance (C_dl_) values estimated from the corresponding CV plots. Figure S6: UOR current density vs. Vcell profile for the overall urea-splitting electrolyzers. Table S1: Comparison of UOR performance for the Cu/Fe-MOF/NF with respect to reported high-performance UOR electrocatalysts. References [64,65,66,67,68,69,70,71,72,73,74,75] were cited in Supplementary Materials.
