# Supplementary material for: Bimetallic Cu/Fe MOF-Based Nanosheet Film via Binder-Free Drop-Casting Route: A Highly Efficient Urea-Electrolysis Catalyst
Source: Nanomaterials (Basel). 2022 Jun 3;12(11):1916. doi: 10.3390/nano12111916 (PMC9182062; doi:10.3390/nano12111916)
Supplement: Supplementary file 1 [file nanomaterials-12-01916-s001.zip › nanomaterials-1723010-supplementary.pdf]

nanomaterials

Supplementary Materials

Bimetallic Cu/Fe Metal-Organic Framework-Based Nanosheet-film via Binder-Free Drop-Casting Route: A Highly Efficient Urea-Electrolysis Catalyst Demonstrating an Ultra-High Current

Supriya A. Patil [1], Nabeen K. Shrestha [2,*], Akbar I. Inamdar [2], Chinna Bathula [3], Jungwon Jung [1], Sajjad Hussain [1], Ghazanfar Nazir [1], Mosab Kaseem [1], Hyunsik Im [2] and Hyungsang Kim [2]

[1] Department Nanotechnology and Advanced Materials Engineering, Sejong University, Seoul 05006, Korea; supriyaapatil11@gmail.com (S.A.P.); jwjung@sejong.ac.kr (J.J.); shussainawan@gmail.com (S.H.); gnazir@sejong.ac.kr (G.N.); mosabkaseem@sejong.ac.kr (M.K.)
[2] Division of Physics and Semiconductor Science, Dongguk University, Seoul 04620, Korea; akbarphysics2002@gmail.com (A.I.I.); hyunsik7@dongguk.edu (H.I.); hskim@dongguk.edu (H.K.)
[3] Division of Electronics and Electrical Engineering, Dongguk University, Seoul 04620, Korea; chinnuchem@gmail.com
* Correspondence: nabeenkshrestha@hotmail.com

Citation: Patil, S.A.; Shrestha, N.K.; Inamdar, A.I.; Bathula, C.; Jung, J.; Hussain, S.; Nazir, G.; Kaseem, M.; Im, H.; Kim, H. Bimetallic Cu/Fe Metal-Organic Framework-Based Nanosheet-film via Binder-Free Drop-Casting Route: A Highly Efficient Urea-Electrolysis Catalyst Demonstrating an Ultra-High Current. *Nanomaterials* **2022**, *12*, 1916. https://doi.org/10.3390/nano12111916

Academic Editor: Hanfeng Liang

Received: 27 April 2022
Accepted: 1 June 2022
Published: 3 June 2022

Publisher's Note: MDPI stays neutral with regard to jurisdictional claims in published maps and institutional affiliations.

Copyright: © 2022 by the authors. Submitted for possible open access publication under the terms and conditions of the Creative Commons Attribution (CC BY) license (https://creativecommons.org/licenses/by/4.0/).

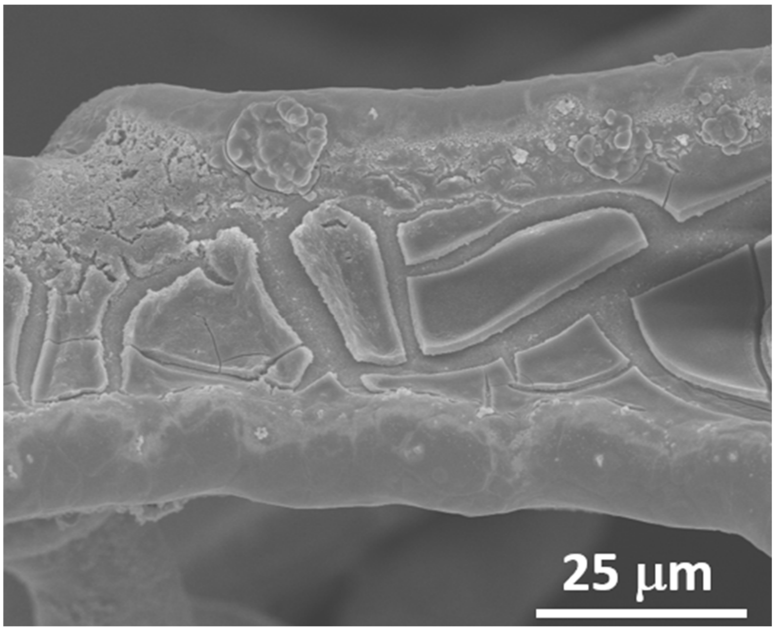

Figure S1. SEM image of the drop-casted Cu/Fe-MOF film on a nickel backbone of the nickel foam substrate, showing the sheet-like structure.

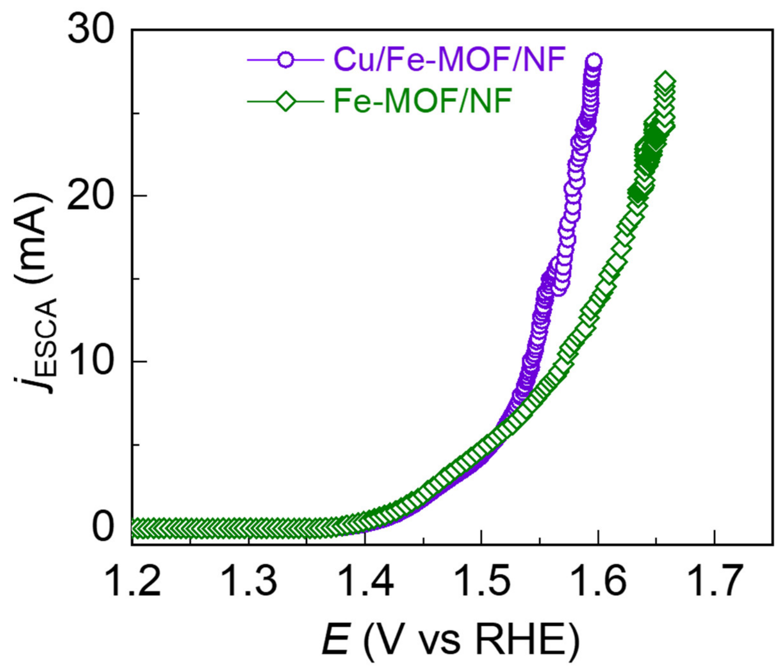

Figure S2. ECSA specific LSV polarization curves obatianed in 1.0 M KOH containing 0.33 M urea by normalizing the geometrical area-based current density by the ECSA.

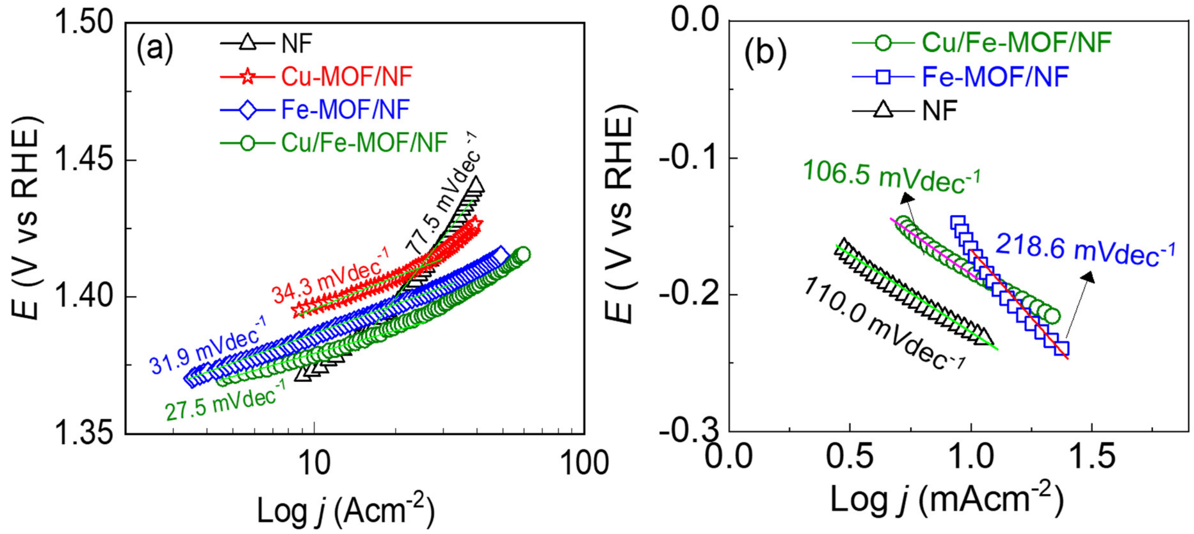

Figure S3. (a) Tafel slopes for UOR extracted from the corresponding anodic LSV polarization curves of "Figure 5a", and (b) Tafel slopes for HER extracted from the corresponding cathodic LSV polarization curves of "Figure 5d".

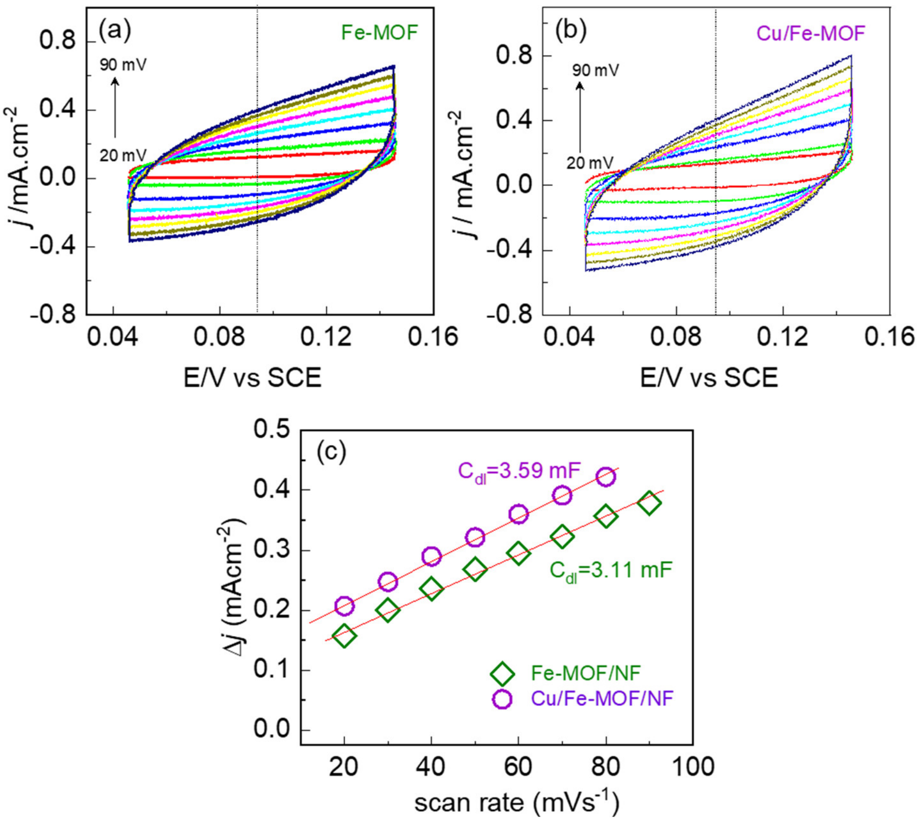

Figure S4. (a-b) CV plots of the Fe-MOF/NF and Cu/Fe-MOF/NF electrodes, respectively in the non-faradaic region in 1.0 M KOH containing 0.33 M urea at various scan rates ranging from 20 to 90 mV s^{-1}. (c) double-layer capacitance (C_{dl}) values estimated from the corresponding CV plots.

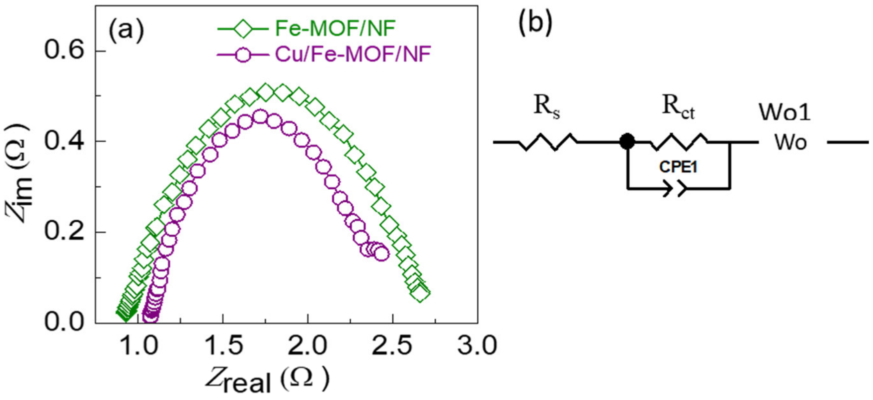

(c)

Element	Fe-MOF/NF	Cu/Fe-MOF/NF
R_s (Ω)	0.93	1.30
R_{ct} (Ω)	1.77	1.24
CPE1 (μF)	0.67	0.68
Z_w (Ω)	0.43	0.41

Figure S5. Electrochemical impedance spectroscopy of the Fe-MOF/NF and Cu/Fe-MOF/NF electrodes. (a) Nyquist plots measured at 1.51 V (vs. RHE), (b) equivalent circuit, and (c) EIS parameter extracted from the Nyquist plots.

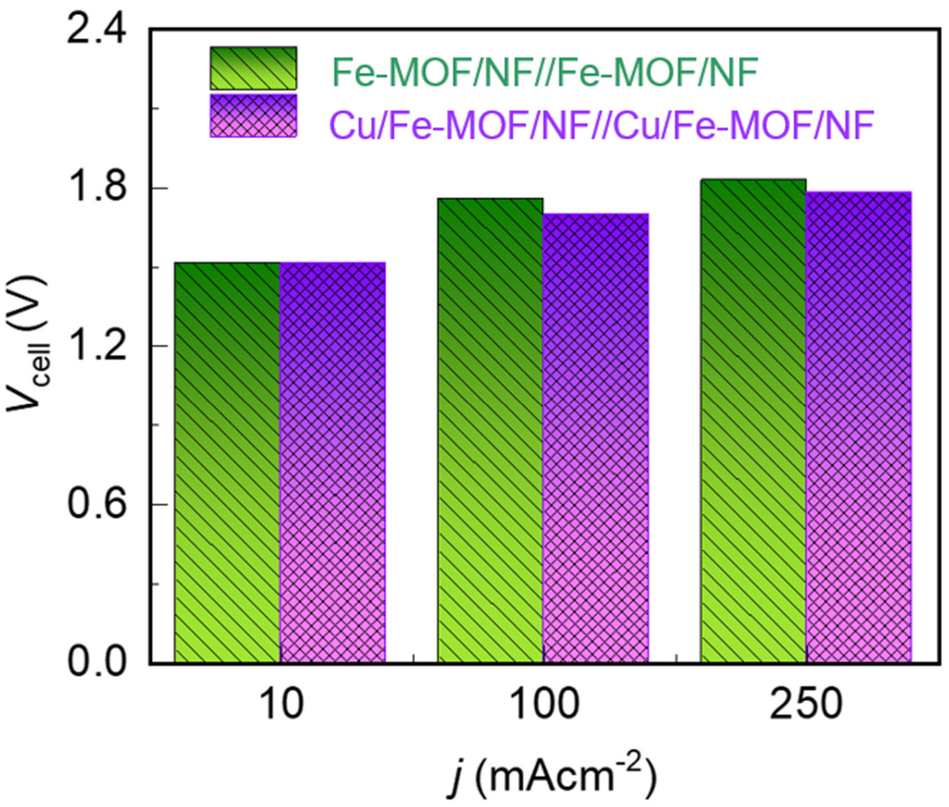

Figure S6. UOR current density vs V_{cell} profile for the overall urea splitting electrolyzers consisting of symmetrical anode and cathode in 1.0 M KOH solution containing 0.33 m urea.

Table S1. Comparison of UOR performance for the Cu/Fe-MOF/NF with respect to reported high-performance UOR electrocatalysts.

	**Electrode materials**	**Electrolyte**	***j* (mAcm^{-2})**	**Cell potential (V) vs RHE**	**Referances**
	Cu/Fe-MOF/NF	1.0 M KOH + 0.33 M urea	100<br>500<br>1000<br>1500<br>2500	1.43<br>1.51<br>1.54<br>1.57<br>1.59	**This work**
	Fe-MOF/NF	1.0 M KOH + 0.33 M urea	100<br>500<br>1000<br>1500<br>2500	1.43<br>1.53<br>1.59<br>1.63<br>NA	**This work**
1	NiFeRh-LDH	1.0 M KOH + 0.33 M urea	500	1.55	*Appl. Catal. B Environ.* **2021**, *284*, 119740
2	NiS@Ni_2S/$NiMoO_4$	1.0 M KOH + 0.5 M urea	100<br>450	1.46<br>1.78	*J. Mater. Chem. A* **2020**, *8*, 18055–18063
3	NiFeCo-LDH nanosheets	1.0 M KOH + 0.33 M urea	170	1.55	*ACS Sustain. Chem. Eng.* **2019**,*7* 10035-10043

4	NFHC	1.0 M KOH + 0.5 M urea	100	1.40	*Sci. Rep.* **2019**, *9*, 1–11
5	Ni metal-organic framework	1.0 M KOH + 0.33 M urea	65	1.50	*Chem. electrochem* **2018**,5 2795-2807
6	V_0-rich-$CoMoO_4$/NF	1.0 M KOH + 0.5 M urea	100	1.51	*Appl. Catal. A Gen.* **2020**, *602* , 117670.
7	NC-PB@CNT	1.0 M KOH + 0.33 M urea	100	1.41	*Chem. Eng. J.* **2021**, *426*, 130773
8	$NiFe(OH)_2$-SD/NF	1.0 M KOH + 0.33 M urea	100	1.52	*J. Colloid Inter. Sci.* **2019**, *557* 10-17
9	NiRh/C	1.0 M KOH + 0.33 M urea	183	1.50	*Electrochim. Acta* **2019**,297 715-724
10	Co_3O_4@Co_2P4O_{12}-300	1.0 M KOH + 0.5 M urea	100	1.60	*Int. J. Hydrogen Energy*, **2019**, *44*, 24705-24711
11	Ni_2P/Fe_2P/NF	1.0 M KOH + 0.5 M urea	100	1.45	*J. Colloid Interface Sci.*, **2019**, *541*, 279-286.
12	CE-NiFe/NF	1.0 M KOH + 0.33 M urea	100	1.5	*Electrochim. Acta*, **2017**, *227*, 210-216
13	NiIr-MOF/NF	1.0 M KOH + 0.5 M urea	100<br>300	1.349<br>1.350	*Chem. Commun.* **2020**, *56*, 2151–2154
14	NiMoO-Ar/NF	1.0 M KOH + 0.5 M urea	100<br>300	1.42<br>1.52	*Energy Environ. Sci.* **2018**, *11*, 1890–1897
15	CoS_2-MoS_2/NF	1.0 M KOH + 0.5 M urea	100<br>350	1.33<br>1.36	*Adv. Energy Mater.* **2018**, *8*, 1–8.

References

1. Sun, H.; Zhang, W.; Li, J.G.; Li, Z.; Ao, X.; Xue, K.H.; Ostrikov, K.K.; Tang, J.; Wang, C. Rh-Engineered Ultrathin NiFe-LDH Nanosheets Enable Highly-Efficient Overall Water Splitting and Urea Electrolysis. *Appl. Catal. B Environ.* **2021**, *284*, 119740, doi:10.1016/j.apcatb.2020.119740.
2. Sha, L.; Liu, T.; Ye, K.; Zhu, K.; Yan, J.; Yin, J.; Wang, G.; Cao, D. A Heterogeneous Interface on NiS@Ni3S2/NiMoO4heterostructures for Efficient Urea Electrolysis. *J. Mater. Chem. A* **2020**, *8*, 18055–18063, doi:10.1039/d0ta04944a.
3. Babar, P.; Lokhande, A.; Karade, V.; Pawar, B.; Gang, M.G.; Pawar, S.; Kim, J.H. Bifunctional 2D Electrocatalysts of Transition Metal Hydroxide Nanosheet Arrays for Water Splitting and Urea Electrolysis. *ACS Sustain. Chem. Eng.* **2019**, *7*, 10035–10043, doi:10.1021/acssuschemeng.9b01260.
4. Feng, Y.; Wang, X.; Dong, P.; Li, J.; Feng, L.; Huang, J.; Cao, L.; Feng, L.; Kajiyoshi, K.; Wang, C. Boosting the Activity of Prussian-Blue Analogue as Efficient Electrocatalyst for Water and Urea Oxidation. *Sci. Rep.* **2019**, *9*, 1–11, doi:10.1038/s41598-019-52412-1.
5. Maruthapandian, V.; Kumaraguru, S.; Mohan, S.; Saraswathy, V.; Muralidharan, S. An Insight on the Electrocatalytic Mechanistic Study of Pristine Ni MOF (BTC) in Alkaline Medium for Enhanced OER and UOR. *ChemElectroChem* **2018**, *5*, 2795–2807, doi:10.1002/celc.201800802.
6. Liu, Z.; Teng, F.; Yuan, C.; Gu, W.; Jiang, W. Defect-Engineered CoMoO4 Ultrathin Nanosheet Array and Promoted Urea Oxidation Reaction. *Appl. Catal. A Gen.* **2020**, *602*, 117670, doi:10.1016/j.apcata.2020.117670.
7. Patil, S.A.; Cho, S.; Jo, Y.; Shrestha, N.K.; Kim, H.; Im, H. Bimetallic Ni-Co@hexacyano Nano-Frameworks Anchored on Carbon Nanotubes for Highly Efficient Overall Water Splitting and Urea Decontamination. *Chem. Eng. J.* **2021**, *426*, 130773, doi:10.1016/j.cej.2021.130773.
8. Babar, P.; Lokhande, A.; Karade, V.; Lee, I.J.; Lee, D.; Pawar, S.; Kim, J.H. Trifunctional Layered Electrodeposited Nickel Iron Hydroxide Electrocatalyst with Enhanced Performance towards the Oxidation of Water, Urea and Hydrazine. *J. Colloid Interface Sci.* **2019**, *557*, 10–17, doi:10.1016/j.jcis.2019.09.012.

9. Mirzaei, P.; Bastide, S.; Dassy, A.; Bensimon, R.; Bourgon, J.; Aghajani, A.; Zlotea, C.; Muller-Bouvet, D.; Cachet-Vivier, C. Electrochemical Oxidation of Urea on Nickel-Rhodium Nanoparticles/Carbon Composites. *Electrochim. Acta* **2019**, *297*, 715–724, doi:10.1016/j.electacta.2018.11.205.
10. Du, X.; Zhang, X. Dual-Functional Co3O4@Co2P4O12 Nanoneedles Supported on Nickel Foams with Enhanced Electrochemical Performance and Excellent Stability for Overall Urea Splitting. *Int. J. Hydrogen Energy* **2019**, *44*, 24705–24711, doi:10.1016/j.ijhydene.2019.07.228.
11. Yan, L.; Sun, Y.; Hu, E.; Ning, J.; Zhong, Y.; Zhang, Z.; Hu, Y. Facile In-Situ Growth of Ni 2 P/Fe 2 P Nanohybrids on Ni Foam for Highly Efficient Urea Electrolysis. *J. Colloid Interface Sci.* **2019**, *541*, 279–286, doi:10.1016/j.jcis.2019.01.096.
12. Wu, M.S.; Jao, C.Y.; Chuang, F.Y.; Chen, F.Y. Carbon-Encapsulated Nickel-Iron Nanoparticles Supported on Nickel Foam as a Catalyst Electrode for Urea Electrolysis. *Electrochim. Acta* **2017**, *227*, 210–216, doi:10.1016/j.electacta.2017.01.035.
13. Xu, Y.; Chai, X.; Ren, T.; Yu, S.; Yu, H.; Wang, Z.; Li, X.; Wang, L.; Wang, H. Ir-Doped Ni-Based Metal-Organic Framework Ultrathin Nanosheets on Ni Foam for Enhanced Urea Electro-Oxidation. *Chem. Commun.* **2020**, *56*, 2151–2154, doi:10.1039/c9cc09484a.
14. Yu, Z.-Y.; Lang, C.-C.; Gao, M.-R.; Chen, Y.; Fu, Q.-Q.; Duan, Y.; Yu, S.-H. Ni–Mo–O Nanorod-Derived Composite Catalysts for Efficient Alkaline Water-to-Hydrogen Conversion via Urea Electrolysis. *Energy Environ. Sci.* **2018**, *11*, 1890–1897, doi:10.1039/C8EE00521D.
15. Li, C.; Liu, Y.; Zhuo, Z.; Ju, H.; Li, D.; Guo, Y.; Wu, X.; Li, H.; Zhai, T. Local Charge Distribution Engineered by Schottky Heterojunctions toward Urea Electrolysis. *Adv. Energy Mater.* **2018**, *8*, 1–8, doi:10.1002/aenm.201801775.
